# Dynamics of vitamin D in patients with mild or inactive inflammatory bowel disease and their families

**DOI:** 10.1186/1475-2891-12-145

**Published:** 2013-11-09

**Authors:** Avigyle Grunbaum, Christina Holcroft, Debra Heilpern, Stephanie Gladman, Barry Burstein, Maryse Menard, Jasim Al-Abbad, Jamie Cassoff, Elizabeth MacNamara, Philip H Gordon, Andrew Szilagyi

**Affiliations:** 1 McGill University School of Medicine, Montreal, Canada; 2 Epidemiology, Jewish General Hospital, Montreal, Canada; 3 Gastroenterology, Jewish General Hospital, 3755 Cote Ste Catherine Rd, Room E177, Montreal, QC, Canada; 4 Department of Medicine, Jewish General Hospital, Montreal, Canada; 5 Clinical Chemistry, Jewish General Hospital, Montreal, Canada; 6 Colorectal Surgery, Jewish General Hospital, Montreal, QC, Canada

**Keywords:** Vitamin D, Kinetics, Inflammatory bowel disease, Families

## Abstract

**Background:**

25(OH) vitamin D levels may be low in patients with moderately or severely active inflammatory bowel diseases (IBD: Crohn’s disease and Idiopathic Ulcerative Colitis) but this is less clear in patients with mild or inactive IBD. Furthermore there is limited information of any family influence on 25(OH) vitamin D levels in IBD. As a possible risk factor we hypothesize that vitamin D levels may also be low in families of IBD patients.

**Objectives:**

To evaluate 25[OH] vitamin D levels in patients with IBD in remission or with mild activity. A second objective is to evaluate whether there are relationships within IBD family units of 25[OH] vitamin D and what are the influences associated with these levels.

**Methods:**

Participants underwent medical history, physical examination and a 114 item diet questionnaire. Serum 25[OH] vitamin D was measured, using a radioimmunoassay kit, (replete ≥ 75, insufficient 50–74, deficient < 25–50, or severely deficient < 25 nmol/L). Associations between 25[OH] vitamin D and twenty variables were evaluated using univariate regression. Multivariable analysis was also applied and intrafamilial dynamics were assessed.

**Results:**

55 patients and 48 controls with their respective families participated (N206). 25[OH] vitamin D levels between patients and controls were similar (71.2 ± 32.8 vs. 68.3 ±26.2 nmol/L). Vitamin D supplements significantly increased intake but correlation with serum 25[OH] vitamin D was significant only during non sunny months among patients. Within family units, patients’ families had mean replete levels (82.3 ± 34.2 nmol/L) and a modest correlation emerged during sunny months between patients and family (r^2^ =0.209 p = 0.032). These relationships were less robust and non significant in controls and their families.

**Conclusions:**

In patients with mild or inactive IBD 25[OH] vitamin D levels are less than ideal but are similar to controls. Taken together collectively, the results of this study suggest that patient family dynamics may be different in IBD units from that in control family units. However contrary to the hypothesis, intra familial vitamin D dynamics do not pose additional risks for development of IBD.

## Background

The discovery of immunoregulatory and antineoplastic functions of vitamin D was followed by research to evaluate its possible pathogenic and therapeutic roles in multiple diseases [1-3]. Epidemiological and observational studies linked increased sunshine and ultraviolet light (UVB, 280 – 315 nm) with diminished risks of many diseases [4-9] including general mortality [7].

The inflammatory bowel diseases (IBD: Crohn’s Disease [CD] and Idiopathic Ulcerative Colitis [UC]) in particular have been noted to have distinct latitudinal distributions [10-12]. While pathogenesis of IBD remains elusive, environmental factors, the host’s intestinal microbial flora and genetic immune modification putatively interact. Multiple predisposing genes, many overlapping between CD and UC have been described [13-15]. However, sunshine and UVB are hypothesized to have beneficial effects [2]. Both diseases have been associated with lower serum 25[OH] vitamin D levels [16,17] but the association is stronger with CD [2,18-23]. Although, not yet proven two trials have been published using supplemental vitamin D for therapy of CD [24]. One suggested a possible reduction in clinical relapse [25]. Evaluation of 25[OH] vitamin D levels in patients with moderate or severe IBD have shown low levels [16,17] but mild or inactive IBD has been studied less frequently.

Vitamin D functions and levels have also been linked with genetic determinants. Vitamin D receptors (VDR) responsible for cell signaling in response to metabolites of vitamin D, have been found within several cell systems. These have been suggested to play a role in IBD [26-28]. In addition 25[OH] vitamin D levels also relate to genetic polymorphisms controlling Vitamin D binding proteins (VDBP) [29,30]. These studies raise questions about possible genetic links determining serum 25[OH] vitamin D levels within families. However, there have been limited reports evaluating intrafamilial relationships of 25[OH] vitamin D levels. An early study from Manitoba in Canada found poor diet led to low 25(OH) vitamin D levels in mothers and infants [31]. Similarly a study from Jordan reported low 25(OH) vitamin D levels in both rachitic infants and their mothers. In this case prolonged breast feeding, poor diet and/or low sun exposure were blamed for the observation [32]. These studies suggest also that low family levels may be determined by environmental issues and may pose a risk for development of diseases.

The aims of this cross-sectional study were to compare serum 25[OH] vitamin D levels in a cohort of IBD patients with mild or inactive disease to a cohort of healthy controls. Furthermore we compared these levels with a first degree family member and to make a second comparison between IBD families to healthy controls and their families. Common clinical variables were looked at and evaluated with respect to their association with levels of 25[OH] vitamin D. We then evaluated the relationships of 25[OH] vitamin D levels within both family units of IBD and controls. We hypothesized that low levels of vitamin D reported in IBD patients may be reflected in family members, thus constituting an additional family risk for IBD.

## Methods

### Patients

This study was carried out at the Jewish General Hospital from March 1, 2009 to April 30, 2011. Approval was obtained from the ethics and review board of the hospital. Patients were invited to participate and recruited from the department’s IBD clinics as well as from gastroenterologists’ practices. Each patient was asked to invite a first degree family member to be evaluated at the same time as the patients. Family members were self chosen by their availability for time to participate. Each participant gave informed signed consent. The diagnosis of Crohn’s disease (CD) or ulcerative colitis (UC) was based on accepted criteria from previous evaluations (history, physical examination, radiology and/or histology) [33]. The recruited patients from clinics were stable (no change in medications for at least 3 months) and had mild or inactive disease). Each patient was assessed at entry into the study and was assigned a clinical index score based on well established criteria. In the case of CD, activity was assessed by the Harvey Bradshaw index [HBI] [34]. For UC the Simple Clinical Colitis Activity Index [SCCAI] was used [35]. In each case both the HBI and SCCAI, the score of ≤ 4 indicates remission. Family members recruited were healthy or had stable (same definition as for stable patients) chronic diseases (hypertension, thyroid hormone replacement or hypercholesterolemia). However diabetes (types 1 and 2), known Celiac disease, other malabsorptive diseases or renal disease were excluded.

A control population was recruited by advertising for health care workers or other employees in the hospital, word of mouth or on university bulletin boards. These were recruited in groups following initial recruitment of patients. The controls were profiled to resemble patients with respect to age, sex, ethnicity and weight. First degree family members of controls (also self chosen by availability of time) were recruited with the same restriction as for patients’ families. There was no attempt to profile family members of controls with family members of patients.

### Interventions

#### Clinical assessment

Participants presented usually as a family unit on the day of the evaluation. All individuals underwent a brief history, physical examination including Body Mass Index (BMI: Kg/M^2^) measurement and a series of blood tests for complete blood count, C-reactive protein, kidney function, biochemistry including calcium albumin and iron studies (ferritin and % saturation). In addition serum vitamin 25 (OH) D levels were measured.

#### Dietary assessment

After brief explanations, all participants completed a 114 item diet questionnaire This questionnaire was presented as a 5-page food frequency questionnaire (FFQ) which was developed and adapted from previously validated questionnaires. The FFQ is a general survey and is not dependent on specific time exposure to the foods. We used a diet limited FFQ to measure daily dietary calcium [87 items] [36], lactose [27 items] and vitamin D intakes [37]. Open-ended questions were included to identify potential contributors and nutrient interactions as dietary supplements and medications. Total daily intake of vitamin D and calcium included both within diet and supplements taken. The main source of vitamin D, calcium and lactose are derived from dairy foods (mostly fluid milk). Lactose is a good surrogate for dairy foods as 12.5 g serving of lactose approximates the content of a 250 ml volume of fluid milk.

Determination of portion sizes was done using 2-dimension food models. A sample of any supplement was requested from participants. A systematic revision of each FFQ was performed by a dietician (SG and MM) and participants were contacted for any clarifications. Double-blinded data entry and analysis was performed using an Excel spreadsheet calculator. Nutrient values extracted from the Canadian Nutrient File 2007 [38] as well as the United States Dietary Association were used as references [39].

#### Vitamin D measurements

Serum 25[OH] vitamin D was measured using an RIA kit as used in the hospital clinical chemistry unit (Immunodiagnostic Systems Ltd. IDS 10 Didcot Way Boldon Business Park, Boldon, Tyne and Wear NE35 9PD). The specificity is 100% for both 25-Hydroxy Vitamin D3 and 24, 25 Hydroxyvitamin D3 and 75% for 25 Hydroxy Vitamin D2.

The interassay precision at 58.7 nmol/L was SD -4.8 and CV of 8.2%. Serum 25(OH) vitamin D levels were reported in nmol/L and daily intake levels in International Units. The North American 25(OH) vitamin D level guidelines were used to classify patients into replete ≥ 75 nmol/L, insufficient 50–74 nmol/L, deficient < 50 nmol/L or severely deficient <25 nmol/L [20,40].

Twenty variables were evaluated to determine univariate relationships to serum 25(OH) vitamin D levels. These variables included age, sex, BMI, race, ethnicity, smoking history, use of tanning salons (relevant in winter only), vacation 1 month prior to blood tests (relevant in winter only), total vitamin D intake, dietary and supplemental vitamin D intake, dairy food intake total calcium intake, dietary and supplemental calcium intake, disease versus no disease, corticosteroid intake, duration of disease, and in CD patients, whether colon or small bowel were affected. Additional effect of UVB was evaluated by assessing month of test and dividing time of testing either into sunny (May – September) or less sunny (October –April) months.

### Statistical analysis

First, descriptive statistics (including means with standard deviations and proportions) were used to describe demographic characteristics, 25(OH) vitamin D serum levels, vitamin D and calcium intakes among patients, controls and their family groups Because these values were skewed in groups, some statistical comparisons were based on log 25(OH) vitamin D and were indicated as such. We used unpaired tests (two-sample *t*-test, chi-square test, or Fisher’s exact test) to compare levels (i.e. serum 25(OH) vitamin D levels, vitamin D and calcium intakes) between patients and controls, and paired tests (paired *t*-test or McNemar’s test for correlated proportions) between patients and their families, and between controls and their families, because we assumed that measured levels within family units were not independent. We then calculated correlations to examine the relationships accounting for seasons between vitamin D intake and 25(OH) vitamin D serum levels between patients and their family group and between controls and their family group, separately by different seasons. We used linear regression with log serum vitamin 25(OH) D as the outcome in univariate and multivariable analysis in order to identify variables that were significantly associated with serum 25(OH) vitamin D levels. Regression analyses were conducted only among the combined sample of patients and controls, because these individuals were considered independent of one another. Finally, we examined and graphed the correlations of log serum vitamin 25(OH) D between patients and family, and between controls and family, separately by seasons.

All tests were two-tailed and statistical significance was accepted at p ≤ 0.05. Analyses were conducted using Stata statistical software (version 8.2, StataCorp, College Station, TX).

## Results

### Participant demographics

A total of 244 people were approached over the 25 month period. All participants lived in Montreal and usually within the same geographic area. Of these 38 were excluded from analysis because they did not meet criteria as described above. Two hundred and six participants remained to be analyzed. Within another 13 participants (6%) 25(OH) vitamin D levels were inadvertently missing and are outlined in tables, leaving 193 for 25 (OH) vitamin D analyses. When possible participants with missing levels were still included for assessment of dietary intakes.

After excluding individuals from the study there remained, 55 patients (21 UC and 34 CD) along with 55 first degree family members and 48 controls together with 48 of their family members. The distribution of family members were as follows: IBD relative family members, mothers 42.6%,daughters 18.5%, sisters 16.7%, and fathers, brothers, sons each 7.3%. Control family members were daughters 29%, sons 23%, mothers 17%, sisters 16.7%, brothers 8%, fathers 6.3%. Demographics of IBD patients are described in Table 1. The majority were female with no significant difference in age between CD and UC patients. The majority in each IBD group were in remission with a mean HBI score of 2.6 ± 1.9 for CD and SCCAI of 2 ± 1.3 for UC. In the CD group however, 7 patients had a mean HBI score of 5.7 indicating mild activity (this represents 20.6% of the CD group and 12.7% of the entire group). Correlation of HBI with serum 25(OH) vitamin D did show a modest inverse correlation (r^2^ = 0.057, r = - 0.24) [data not shown]. These 7 also had elevations of CRP. None of the UC patients demonstrated activity by scores, although 4 had elevated CRP. CRP levels did not correlate well in 10 patients with available serum 25(OH) vitamin D levels [n = 10 patients], (r^2^ = 0.001, r = 0.029) [data not shown].

**Table 1:** Demographics Counts (%) are reported unless otherwise noted

		**Patients**		**Controls and family groups**		
		**Crohn’s disease (n = 34)**	**Ulcerative colitis (n = 21)**	**Patients’ family (n = 55)**	**Healthy control (n = 48)**	**Controls’ family (n = 48)**
**Age**, mean ± SD		39.9 ± 12.3 yr	44.2 ± 13.7 yr	46.5 ± 21.6 yr	39.6 ± 13.8 yr	30.5 ± 18.7 yr
**Sex**: female		21 (61.8%)	13 (61.9%)	42 (76.4%)	38 (79.2%)	30 (62.5%)
**BMI**, mean ± SD		24.2 ± 4.1 kg/m^2^	25.4 ± 3.2 kg/m^2^	24.6 ± 4.6 kg/m^2^	25.7 ± 7.9 kg/m^2^	24.9 ± 6.5 kg/m^2^
**Ethnicity**:	Caucasian*	32 (94.1%)	20 (95.2%)	51 (92.7%)	38 (79.2%)	38 (79.2%)
	Jewish	15 (44.1%)	13 (61.9%)	29 (52.7%)	20 (41.6%)	20 (41.6%)
**Seasons**:	Sunny: May - Sept	15 (44.1%)	10 (47.6%)	28 (50.9%)	21 (43.8%)	22 (45.8%)
	Less Sunny: Oct. - Apr	19 (55.9%)	11 (52.3%)	27 (49.1%)	27 (56.3%)	26 (54.2%)
**Smoking^**		3 (8.8%)	3 (14.3)	5 (9.1%)^	6 (12.5)%	2 (4.2)%
**Regular tanning^**		0	1 (4.8%)	0	1 (2.1)%	1 (2.1)%
**Vacation 1 month pretest^**		4 (11.8%)	0	3 (5.5)%	4 (8.3%)	1 (2.1)%
Activity score**, mean ± SD		2.6 ± 1.9 (20.6% mild active)	2 ± 1.3 (0% active)			
**Disease Duration**, mean ± SD		85.6 ± 140.2 month	39.7 ± 53.5 month			
**Site of disease**	Colon	13 (38.2%)	Pan Colitis: 16 (76.2%)			
	Ileo-colon	12 (35.3%)	Left limit: 4 (19%)			
	Small bowel	9 (26.6%)	Unclear: 1 (5%)			
**Medical**:	5ASA	6 (17.7%)	17 (81%)		0	1 diagnosed with leukemia
	Corticosteroids	4 (11.8%)	4 (19%)			
	Immunomodulators	5 (14.7%)	N1 (4.8%)			
	Biologics	3 (8.8%)	3 (14.3%)			
**Surgical history**:	Colon	0	Colectomy: 1 (4.8%)			
	Colon and/or Small Bowel	11 (32.4%)				
	Ileostomy	3 (8.8%)				

* Chi-square comparison of Caucasian in Patient vs. Controls yields p = 0.019.

^ Missing information on participants ranged from 25-50%.

** Score for Crohn’s based on Harvey Bradshaw Index [34] Inactive ≤ 4.

Score for Ulcerative Colitis based on Simple Clinical Colitis activity Index [35]. Inactive < 4.

More than half of the patients were Jewish and only 6% were non Caucasians. The CD patients had their disease twice as long as the UC patients. The distribution of CD was mainly colon or colon and terminal ileum. None had gastric or duodenal disease based on current history and previous endoscopy or radiological assessment. Of the UC patients 75% had pancolitis and the others had left sided disease only. As expected 5-Amino Salicylic Acid (5ASA) use was common in UC but less than 20% in CD. Immunomodulators and biologics were used in a minority of patients. In the CD group 14 patients underwent at least one previous operation. One patient with UC had a remote colectomy. Table 1 outlines the demographics of families of IBD patients, controls and their families. Similar to the patient’s group the majority of family members were female and there was no significant age difference from patients. Family members of patients were a decade older than family members of controls. Two controls and 2 family members of controls had CRP elevations. Non Caucasians were unevenly distributed in the groups (patients 3/55 [5.5%] and controls 10/48 [20.8%] respectively (Fisher’s p = 0.034).

### Dynamics of vitamin D in groups

#### Serum 25 (OH) vitamin D levels across 4 study groups

The results of measured 25(OH) vitamin D levels and their distributional rank criteria as described in the method section are shown in Table 2. There was no significant difference in levels or distribution of vitamin D classifications between CD and UC patients or between IBD group and controls. One patient with UC had a level of 25(OH) vitamin D < 25 nmol/L (severe deficiency). The mean serum 25(OH) vitamin D levels in the IBD family group was significantly higher than patients’, using paired *t* test for both actual and log values.

**Table 2:** Serum 25[OH] vitamin D by Groups

Group*	**Serum Vit 25(OH) D**	**Replete: ≥ 75**	**Insufficient: 50-74**	**Deficient: < 50**
	**mean ± SD**	**n (%)**	**n (%)**	**n (%)**
IBD Patients	71.2 ± 32.8**	21 (38.2%)	12 (21.8%)	17 (30.9%)
		15(71.4%)	3 (25%)	8 (47.1%)
Crohn’s Disease	71.1 ± 31.1	14 (41.2%)	7 (20.6%)	10 (29.4%)
		9 (64.3%)	2 (28.6%)	5 (50%)
Ulcerative Colitis	71.4 ± 36.3	7 (33.3%)	5 (23.8%)	7 (33.3%)
		6 (85.7%)	1 (20%)	3 (42.9%)
Patients’ Family	82.3 ± 34.2	27 (49.1%)	18 (32.7%)	7 (12.7%)
		13 (48.2%)	5 (27.8%)	1 (14.3%)
Healthy Control	68.3 ± 26.2	15 (31.3%)	19 (39.6%)	11 (22.9%)
		7 (46.7%)	11 (57.9%)	4 (36.4%)
Controls’ Family	69.9 ± 27.6	19 (39.6%)	16 (33.3%)	11 (22.9%)
		8 (88.9%)	5 (31.3%)	1 (9.1%)

Levels and categories of serum 25[OH] vitamin D in nmol/L overall and by patient, control and their respective families are shown. The lower values represent the number of participants in each category who consumed supplemental vitamin D and the percentage of the category in each group. Based on (n = 193) available serum levels.

* Because of some missing data on serum 25[OH] vitamin D levels, percentages do not sum to 100%. Missing data were as follows:

5 from IBD.

3 from Family IBD.

3 from Control.

2 from Family Control.

** patient vs. family: p = 0.0332 (paired *t* test) or p = 0.0164 (for log values).

#### Association of vitamin D intake, calcium intake, and season with serum 25(OH) vitamin D levels

Description of daily vitamin D (total = dietary + supplements) and calcium (total = dietary + supplements) intake in the four groups is shown in Table 3. The number of participants who consumed vitamin D supplements in each study group is also reported. Controls consumed more daily vitamin D, without statistically significant differences in intake between patients and controls. However, supplemental vitamin D daily intake significantly increased intake, to a range of 1100 – 1350 (Table 3). Although higher than the RDA, these do not approach the tolerable upper limit (UL) for vitamin D of 4000 IU for children > 9 yrs. and adults. Patients and controls had similar frequency of supplementary intake of vitamin D: patients: 53%, IBD family: 36%, healthy controls: 48%, controls family: 31%.

**Table 3:** Intake of total, dietary or supplemental vitamin D and calcium by groups

	**IBD Patients**	**Patients’ family N = 55**	**Controls**	**Controls’ family N = 48**
	**N =55**		**N = 48**	
**Dietary Vit D intakes in each group, n**	54	54	46	48
Mean ± SD IU	371 ± 333	527 ± 803	600 ± 848	666 ± 592
Median	286	260	394	416
**Total Vit D Intakes in each group, n**	54	54	46	48
Mean ± SD	784.4 ± 720	761.3 ± 881.6	917 ± 1028	867 ± 801
Median	599.5	523	590.2	612.
**Total Vit D intakes in Subgroups with Supplemental Vit D, n**	29	20	23	15
Mean ± SD	1110 ± 780*	1116.2 ± 809.7**	1279 ± 857**	1344.9 ± 994.2**
Median	932.4	932.8	1020.8	1191.1
**Total Vit D intakes in Subgroups without Supplemental Vit D, n**	23	34	23	32
Mean ± SD	404.2 ± 409.2	552.5 ± 865.8	554.3 ± 1074.4	648.4 ± 605.3
Median	332.8	291.7	290.4	377.4
**Total Calcium Intakes in each group, n**	54	54	46	48
Mean ± SD	1537.2 ± 755	1687.4 ± 1071.5	1778.7 ± 1127.2	1931.4 ± 1325.6
Median	1479	1404.7	1552.1	1679.5

Vitamin D intake is listed in International Units (conversion to μg is IU/40). Total daily calcium is listed as g/day. Vitamin D intakes were skewed and therefore both mean ± standard deviation and median values are listed. N values for each group represents original number in the groups and n values actual numbers analyzed.

Comparisons of means of groups with and without vitamin D supplements were significant; * p < 0.01, ** p < 0.05.

Table 4 contains average serum 25[OH] vitamin D levels across categories of vitamin D intake with and without supplements, and across seasons, for each study group. Frequency toward replete levels increased but statistical significance was achieved only in patients depending on analysis (mean ± SD, p = 0.03 or mean log vitamin D, p = 0.059).

**Table 4:** Serum 25[OH] vitamin D according to intakes of vitamin D

**Item**	**IBD Patients**	**Patients’ Family N = 55**	**Controls**	**Controls’ Family N = 48**
	**N = 55**		**N = 48**	
**25(OH)D levels in Supplement users, n**	26	19	22	14
Mean ± SD	79.8 ± 36.5*	86.7 ± 24.1	70.2 ± 26.5	74.6 ± 15.9
**25(OH)D levels in non Supplement users, n**	21	32	22	31
Mean ± SD	59.4 ± 25.0	79.9 ± 39.6	67.9 ± 26.1	68.2 ± 31.8
**Seasons:**				
May-Sept, n	23	27	19	21
Mean ± SD	81.6 ± 32.5 **	83.7 ± 32.1	69.5 ± 26.3	73.3 ± 30.9
Oct-April, n	27	25	26	25
Mean ± SD	62.4 ± 31.0	80.8 ± 36.9	67.4 ± 26.6	67.1 ± 24.7

The effects of vitamin D intake on 25[OH] vitamin D with or without supplement or seasons of testing are shown for four groups. All serum 25[OH] vitamin D levels are reported as nmol/L. May –Sept are sunny months, October –April are non sunny months. N values for each group represents original number in the groups and n values actual numbers analyzed.

* The comparison of means ± SD was significant (p = 0.03 compared with patients not taking vitamin D supplements). However comparing log 25[OH] vitamin D the result became nonsignifcant (p = 0.059).

** p = 0.039 compared with patients evaluated in October to April.

Intake of calcium appeared to affect 25[OH] vitamin D levels but this was evident mainly in controls (data not shown). Calcium supplements were used by 58% and 46% of patients and controls respectively (NS). Similarly controls consumed more calcium than IBD patients, but the difference was not statistically significant. Controls did consume about twice as much dairy foods as patients (lactose: Controls, 21.9 g/d, Patients, 11.7 g/d, p = 0.01).

Regarding seasons, serum 25[OH] vitamin D levels in patients only were significantly increased in sunny months May – September compared to less sunny months October – April (Table 4). Among patients and controls combined (N 95) the mean 25(OH) vitamin D level during sunny months.

(N42, May – September) was 76.1 ±30.1 nmol/L while during less sunny months (N53, October- April) the level was 64.8 ±28.7 nmol/L (p = 0.046, for log 25(OH) vitamin D).

Similarly taking a sunny vacation one month prior to testing or using tanning salons resulted in higher levels but there were too few participants for meaningful statistical analysis.

**Figure 1:**
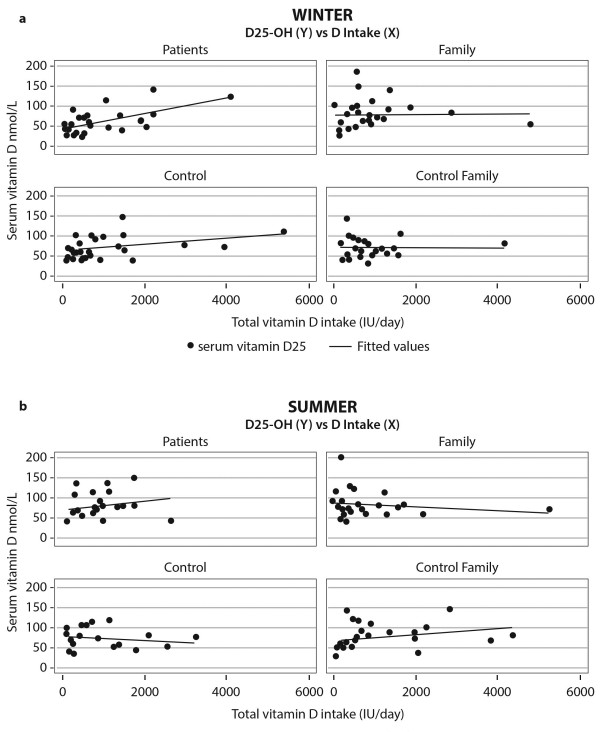
**25[OH] vitamin D levels and the effect of intake in groups by seasons. a**. *Winter*: Patients (N 26) (r^2^ = 0.315, r = 0.561, p = 0.003), Families of patients (N25) (r^2^ < 0.001, r = 0.003, p = 0.990), Controls (N26) (r^2^ = 0.129, r = 0.359, p = 0.072) and Families of controls (N25) (r^2^ < 0.001, r = 0.010, p = 0.961). **b**. *Summer*: Patients (N = 23): (r^2^ = 0.036, r = 0.191, p = 0.384), Families of patients (N26): (r^2^ = 0.024, r = -0.156, p = 0.448), Controls (N18): (r^2^ = 0.018, r = -0.134, p = 0.597) and Families of controls (N21): (r^2^ = 0.092, r = 0.303, p = 0.182).

Correlations between vitamin D intake and serum 25[OH] vitamin D levels are shown in Figure 1a and 1b, separately by seasons and for each study group. It is noted that the association of vitamin D intake with serum levels was more evident during less sunny months and was more robust in patients (r^2^ = 0.315, r = 0.56, p = 0.003) than in controls (r^2^ = 0.13, r = 0.36, p = 0.07). There was no discernable effect of vitamin D intake by seasons in either family group.

#### Univariate and multivariable analyses for serum 25(OH) vitamin D in patients and controls combined

We analyzed 20 individual variables as described in the methods section for univariate association with log serum 25(OH) vitamin D levels. Since there were no significant differences in 25(OH) vitamin D levels between patients and controls, these groups were combined for analyses. Five variables were statistically significant: Jewish ethnicity, vacation one month prior to study testing, total calcium intake, dietary (but not supplemental) calcium, and seasonal measurement of levels. Five other variables reached marginal significance (p < 0.1). These were total vitamin D intake, Caucasian race, BMI, dietary (but not supplemental) vitamin D, and use of corticosteroids by patients.

Caucasians had higher serum 25[OH] vitamin D levels than non Caucasians in both patients and controls [Caucasian patients vs. non-Caucasian patients: 71.7 ± 33.6 vs. 64 ± 18 nmol/L respectively] [Caucasian controls vs. non-Caucasian controls: 72.3 ± 22.8 vs. 54.5 ± 33.3 nmol/L respectively]. In IBD and family groups only 3 non Caucasians were included contributing little to mean values of serum vitamin D. In the control group, 10 participants and 9 participants in their family group were non Caucasians. Comparison of mean levels of 25[OH] vitamin D for Caucasians versus non Caucasians was of borderline significance in the controls (p = 0.0575) but was more substantial in the control family (p = 0.003). Exclusion of all non Caucasians did raise the mean 25[OH] vitamin D level to 75.6 ng/L). Comparison of means in Caucasian patients with Caucasian controls after removal of non Caucasians still showed no significant differences [71.7 ± 33.6 vs. 72.3 ± 22.8 (SD) nmol/L respectively NS].

In multivariable analysis seasonal testing, Jewish ethnicity, intake of dietary calcium and intake of total vitamin D remained in the model (n = 91).

#### Dynamics of Vitamin D within family units

**Figure 2:**
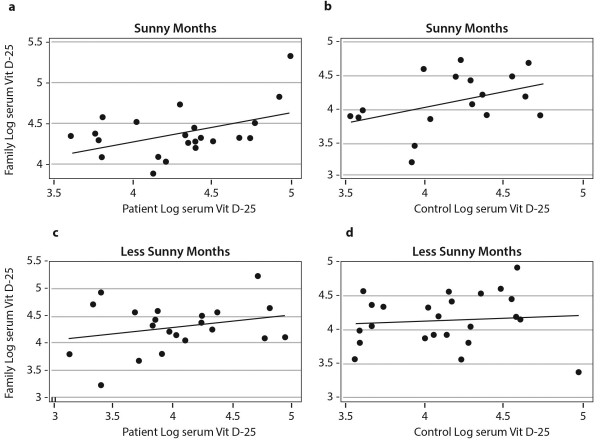
**Relationships of serum 25[OH] vitamin D levels within families by seasons. a**. Correlations, slopes and p-values for plots of log serum VitD-25 of Patients vs. Families of patients in Summer (N22) (r^2^ = 0.2098, r = 0.458, p = 0.032): **b** winter (N22) (r^2^ = 0.0672, r = 0.259, p = 0.24), **c** Controls vs. Families of controls in summer (N18) (r^2^ = 0.1969, r = 0.444, p = 0.065), **d** in winter (N25) (r^2^ = 0.0068, r = 0.082, p = 0.6955).

Overall there was a modest non significant correlation in log 25[OH] vitamin D levels within families (patients and family r^2^ = 0.06, r = 0.24; p = 0.1 control and family r^2^ = 0.08, r = 0.29; p = 0.06). When family units were separated by sunny or less sunny season of testing, the levels of log values between patients and their families became statistically significant (r^2^ = 0.209, r = 0.46, p = 0.032 in sunny months vs r^2^ = 0.0672, r = 0.26, p = 0.244 less sunny months) (Figure 2a and 2c respectively). This altered relationship was weaker between controls and their families (r^2^ = 0.197, r = 0.44, p =0.065 more sunny months, r^2^ = 0.007, r = 0.08, p = 0.695 less sunny months) (Figure 2b and 2d).

Among the four groups, IBD family members had the highest mean replete 25[OH] vitamin D levels (82.3 ± 34.2 nmol/L) and highest frequency in this category (49.1%, Table 2).

Comparison of the distribution of deficiency categories was not significantly different between patient and controls (chi-square p = 0.164), but was significantly different between IBD patients and their family members (McNemar’s p = 0.0102), indicating a more deficient state in the patients.

## Discussion

The main outcomes of this study are as follows. Firstly, approximately 2/3 of individuals within the group of IBD patients with mild or inactive disease have insufficient or deficient 25[OH] vitamin D levels. However this is similar to that found in healthy controls. We confirm seasonal variation in 25[OH] vitamin D levels. Measurement of serum 25[OH] vitamin D during sunny months significantly affected patients levels. Overall consumption of vitamin D supplements increased patient’s serum levels (marginal statistical significance). Families of patients or controls and their families appeared to be less affected by these variables. The IBD families as a group, show higher mean 25(OH) vitamin D level and more often achieve replete status than patients. A modest but significant relationship with patients’ levels becomes evident during sunny months.

Our definitions of the distributions for 25[OH] vitamin D have an influence over the proportion of replete and insufficient number in groups. The controversy over acceptable levels of 25[OH] vitamin D continue and are accentuated by the recent decision of the Institute of Medicine (IOM) to endorse the 50 nmol/L as optimal value for skeletal health [41]. If we accept the IOM definition, 2/3 of patients and 3/4 of controls remain replete. However, there is controversy whether levels of 25[OH] vitamin D acceptable for skeletal health are also applicable to autoimmune or other diseases and the IOM decision was criticized [42]. Our hospital accepts 25[OH] vitamin D distributions at the higher levels as defined herein. In this way about 60% of IBD patients and 2/3 of controls remain insufficient. Other studies, including mostly active cases have reported lower levels in IBD [16,17,20-22], but others do report results similar to our study [43,44]. In the current study, although only a few patients with CD had modestly elevated HBI scores, there was a weak inverse correlation with serum 25(OH) vitamin D levels, suggesting that perhaps active disease is the cause (or effect of) lower serum 25(OH) vitamin D in such cases.

### The impact of seasons and vitamin D intake

Evaluating the most relevant variables affecting serum 25[OH] vitamin D levels, MVA ranked sunshine as the most influential on serum levels. The effect of seasons on serum 25[OH] vitamin D has been previously described [22]. Seasonal variation altered serum levels and the effect of vitamin D intake on levels. The effects were more evident in patients as sunshine compensated for intake in sunny months while vitamin D intake affected 25[OH] vitamin D levels in non sunny months. Still these effects did not as a whole compensate enough to achieve uniform replete levels.

In the current study patients who consumed vitamin D supplements had higher levels than those who relied solely on diet, but levels still did not reach desired values. About half the patients and controls consumed supplements but almost 60% did not achieve replete levels. Nevertheless the intake of supplements was disappointing. Only in the patient’s group was there an apparent important increase in levels seconday in vitamin D supplemental intake but did not reach conventional statistical significance. The explanation for the effect on patients is not clear, especially in the light of a recent finding that at least CD patients may have a 35% less efficiency in absorption of vitamin D [45].

Therefore, larger quantities of vitamin D intake may be needed in general and in winter in particular. At least 1200 IU/d (15 μg) is required for replete levels [25]. However, Aloia suggested that 3200 IU/d should be used for at least 2 months and a simulated model found that 4600 IU/d consistently achieved the 75 nmol/L mark [40].

The MVA also ranked Jewish ethnicity and dietary calcium as important determinants of serum vitamin D levels in this study. As explained in the results Jewish ethnicity frequency was similar in both patients and control groups. Their serum 25[OH] vitamin D levels were not significantly different from non Jewish groups but was more evident in the control group.

Dietary calcium was significantly related to serum vitamin D levels but a relationship was only observed in controls. The reason for this is not clear. It is possible that this variable reflected the increased dairy food consumption in controls with increased vitamin D added to such foods. Calcium intake can increase 25[OH] vitamin D levels, when the source of both is from milk but not other sources [46].

### The impact of intrafamilial dynamics

Contrary to our expectations IBD family members as a group had the highest rather than lowest mean 25[OH] vitamin D levels. The levels in patient’s families were significantly higher than those in patients. The difference between them was significantly greater than the difference noted between controls and their families. Furthermore a modest but significant correlation in the IBD family unit emerged during sunny months which was less evident in the control family unit.

The reason(s) for these findings is (are) not clear. The possibilities include chance, because of relatively few participants in each group. Our control group may not have been as healthy as expected. 25(OH) vitamin D results of current controls were however, similar to those reported by others [43,44] and were similar to another healthy control group reported from Montreal [47].

The inclusion of more non Caucasians in the control groups may have limited the 25[OH] vitamin D levels in the controls and their families. Non Caucasians may have lower 25[OH] vitamin D levels [48] However the numerical advantage of the IBD families was only modestly diminished by removal of the non Caucasians from controls. Furthermore the consistent relationships, observed in patients and the family unit rather than controls and their family unit, reduces somewhat the role of chance or the inclusion of excessive non Caucasians in the latter family unit. Still, it is possible that intra family dynamics are different between Caucasians and non Caucasians and this may have influenced intra family results.

In view of intra IBD family unit observations proposal of a genetic link unique to IBD patients and their families is tempting. However, failure to recruit both parents and inclusion of random single family members instead, dilutes ability to detect firm connections. Other undetected variables may also have contributed.

### Limitations of study

Limitations in this study need to be considered. Firstly there are a paucity of participants and lack of homogeneity of the IBD group. The use of the combination of IBD patients rests on the similar 25[OH] vitamin D levels found in both groups. A second limitation is the use of clinical indexes to define clinical status of patients. Although, well recognized as markers of activity the HBI or SCCI do not preclude false judgment of remission or activity. The presence of symptoms may indicate irritable bowel related symptoms or those secondary to previous surgery (eg. diarrhea). In conjunction with this notion is that we evaluated patient’s status on a single blood test measurement, which does not allow any longitudinal assessment of clinical course. A third limitation is the absence of data in different categories. Most of these were inadvertent but data that were not available were not used to derive effects on serum vitamin D. In other situations however available data were used to answer questions pertaining to an outcome. For example intake of vitamin D was used to derive that particular information but was not used to relate to serum levels if those values were unavailable. Another limitation was in the way the data were analyzed (combination of patients and controls for effects of variables) may have precluded demonstration of disease effect on 25[OH] vitamin D levels. With the exception of a weak impact of corticosteroids, none of the previously shown effects of IBD on 25[OH] vitamin D levels were found [20,49,50]. However, the reason for lack of disease effect, may also relate to the clinical stability of our patients which mostly included inactive or mildly active disease. This suggests that disease activity is more likely an important determinant of serum 25[OH] vitamin D.

## Conclusions

We report on 25[OH] vitamin D levels in a practical clinical setting for stable IBD patients predominantly in remission. Seasonal effects and the intake of vitamin D supplements appeared to be more evident in the IBD family unit largely driven by patients reactions.

Taken together this study suggests different vitamin D dynamics in patient’s family units than in control’s family units. The explanation for these observations is not evident from this study. However, it does suggest that further evaluation is needed to determine optimal vitamin D intake to uniformly achieve replete levels in IBD. Furthermore we demonstrate that IBD family vitamin D dynamics do not increase risk for these diseases. Further work would be needed to determine whether these dynamic differences can be reproduced.

## Abbreviations

IBD: Inflammatory bowel disease; CD: Crohn’s disease; UC: Ulcerative colitis; UVB: Ultraviolet light B (280 – 315 nm); BMI: Body mass index (Kg/m^2^); FFQ: Food frequency questionnaire; CV: Coefficient of variation; IOM: Institute of medicine; IU: International units; MVA: Multivariable analysis.

## Competing interests

The authors declare that they have no competing interests.

## Authors’ contributions

CH: Design of Statistical approach, analysis of data, writing of manuscript. AG: Carrying out clinical portions of the study, Data entry, and discussions about the topic, presentation of abstract ACG Washington, correction and approval of manuscript. DH, BB, JC and JA; all participated in discussions of the topic, carrying out clinical portions of the study, data entry reviewed and approved the final manuscript. SG and MM: Design of diet questionnaires and pictures of portion sizes. Analysis of lactose, calcium and vitamin D content of intakes. Data entry and synthesis for statistical calculations. EM: responsible for serum vitamin D analysis, discussions of the topic. Advice on aspects of writing the manuscript, review and approval of final manuscript. PHG: Discussions on design of the study, review of presentations at meetings and review and approval of final manuscript. AS: Design of study, Initiation of project. Writing of manuscript. All authors approve the final manuscript.
